# Femoral Metastasis from Penile Carcinoma: Report of 2 Cases

**DOI:** 10.1155/2015/583851

**Published:** 2015-10-22

**Authors:** Laura Braumann, Panagiotis Tsagozis, Rikard Wedin, Otte Brosjö

**Affiliations:** ^1^Department of Orthopedics and Orthopedic Surgery, General Hospital Oberndorf, Paracelsusstraße 37, Oberndorf, 5110 Salzburg, Austria; ^2^Section of Orthopedics, Department of Molecular Medicine and Surgery, Karolinska Institute, 17176 Stockholm, Sweden; ^3^Department of Orthopaedics, Karolinska University Hospital, 17176 Stockholm, Sweden

## Abstract

*Purpose*. Penile cancer rarely gives symptomatic skeletal metastases. *Methods*. We present 2 patients with squamous carcinoma of the penis who were surgically treated for metastases in the femur. *Results*. Both patients had pathological fractures and were operated on. In one case, the skeletal metastasis preceded any lymphatic spread of the disease, suggesting early haematogenous dissemination. *Conclusions*. Endoprosthetic reconstruction resulted in pain relief and restored the ambulatory capacity. Clinicians should be aware of the possibility for symptomatic bone metastases with a risk for pathological fracture in patients with penile cancer.

## 1. Introduction

Primary penile carcinoma constitutes approximately 1% of male cancers worldwide, squamous cell carcinoma accounting for 95% of cases. The disease is usually localised, with metastases affecting <3–5% of patients [1]. The metastatic pattern is fairly predictable: Superficial and deep inguinal nodes are the first to be involved and iliac lymph nodes are affected later on. Most common sites for distal metastases are lymph nodes, liver, and lungs. Bone metastases are rare, mostly localised in the axial skeleton [2–5]. However, indolent skeletal metastatic foci are probably common in patients with advanced disease [6]. On the other hand, appendicular skeletal metastasis from penile squamous cell carcinoma has been reported only once in the medical literature, in the case of a patient with disseminated metastatic disease that presented with a pathological fracture of the humerus [7].

Generally, metastatic disease of the appendicular skeleton results in significant morbidity. It requires operative treatment in order to facilitate weight bearing and minimize pain in contrast to metastases in the axial skeleton, which can often be treated with radiotherapy. It is therefore important that physicians have a high degree of awareness and are familiar with the appropriate procedures for diagnosis and treatment. We report 2 cases of femoral metastases with pathological fractures in patients with penile carcinoma, who were successfully treated with endoprosthetic reconstruction. In one case, the metastasis occurred before any lymphatic dissemination of the disease.

## 2. Case Presentation

**Figure 1:**
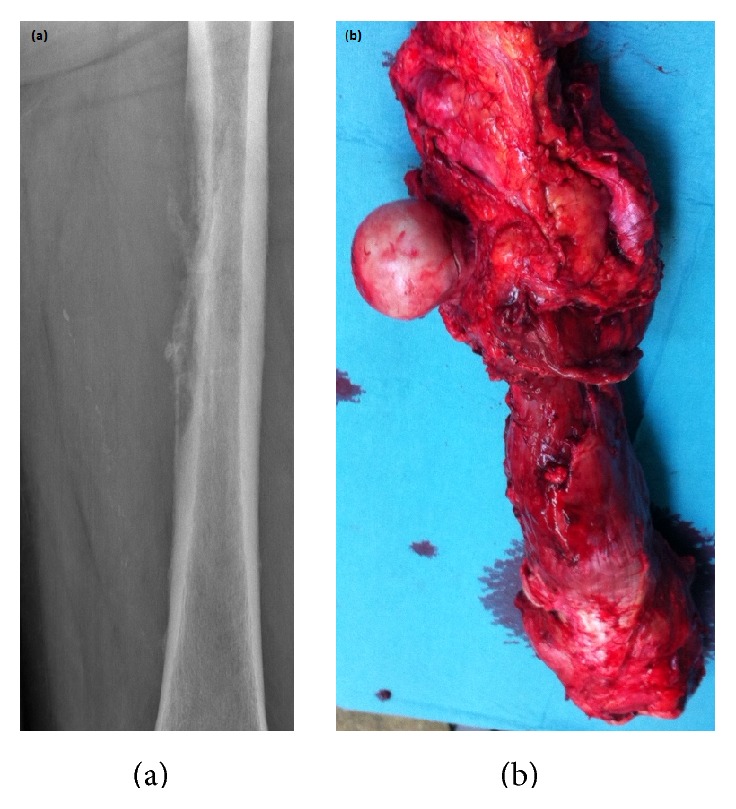
Plain X-ray of the left femur in a patient with penile cancer without any evidence of lymphatic spread reveals a lytic metastasis in the shaft of the bone (a). The left femur excised en bloc prior to reconstruction with a tumor endoprosthesis (b).

A 71-year-old man who was previously diagnosed with stage II disease of a high-grade penile carcinoma presented with a single diaphyseal femoral metastasis manifesting itself as a pathological diaphyseal fracture half a year later. The patient had undergone a radical partial penectomy and excision of sentinel nodes that proved to be free of any signs of metastatic spread. He was initially treated with intramedullary nail osteosynthesis and received adjuvant radiotherapy at the referring hospital. The patient was referred to our centre 3 months after surgery because of severe pain, swelling, and discomfort of the leg. A CT scan showed widespread, almost total destruction of the femur with acetabular and soft tissue involvement (Figure 1(a)).

**Figure 2:**
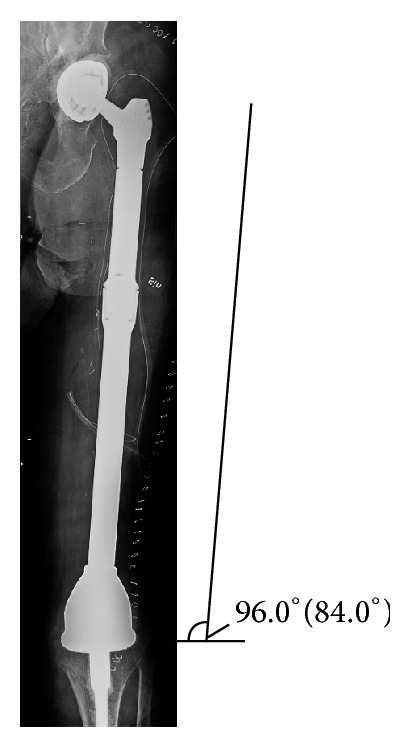
Reconstruction of the whole femur with a modular tumor endoprosthesis (METS modular implant system, Stanmore Implants, Elstree, WD6 3SJ, UK). Notice good placement of the prosthetic parts and proper alignment with the tibia.

Due to the extensive tissue involvement, en bloc excision of the soft tissue component and engaged bone (Figure 1(b)) and subsequent total femur reconstruction with a modular tumor endoprosthesis were performed (Figure 2). The pathology report confirmed the metastatic involvement of the proximal femur. Postoperative course was uneventful; at the latest follow-up the patient was ambulatory, with a good quality of life, and without any signs of relapse of the primary cancer or any signs of metastatic spread.

**Figure 3:**
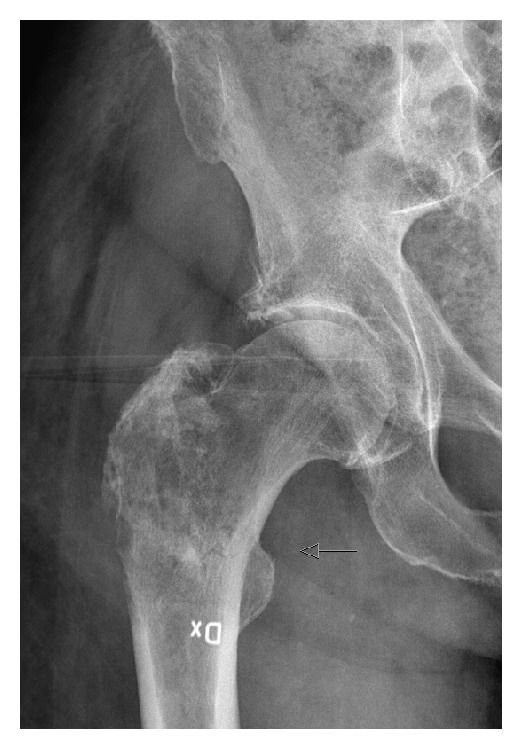
Plain X-ray of the right femur in a patient with advanced metastatic penile cancer reveals a lytic metastasis in the subtrochanteric region and a nondisplaced fracture (arrow).

A 76-year-old patient with stage IV disease (abdominal lymph node metastases) presented with pain in the right thigh. He had been diagnosed with a metastasis in the proximal femur 9 years after his primary tumor treatment and was primarily treated with radiotherapy. There was however progress of the local symptoms and radiology revealed a nondisplaced pathological fracture in the subtrochanteric region of the femur (Figure 3). There was also evidence of hypercalcemia, probably due to the osteolytic component of the tumor. After reconstruction with a cemented hemiarthroplasty, recovery was uneventful and the patient could directly after the operation resume ambulation.

## 3. Discussion

Regional lymph node involvement occurs early in penile carcinoma but remains as a locoregional process for a long time. Distant metastases are uncommon, predominately affecting distant lymph nodes, and are generally accompanied by regional lymph node metastases. The rare haematogenous spread to liver, lungs, brain, skin, and bone is associated with a worse prognosis. Metastases to the spine and pelvis have been sporadically described in the literature [2–5]. However, underdiagnosis or underreporting might be frequent, and the exact incidence of these findings is uncertain. Indeed, in 10 autopsy specimens of patients with advanced disease, bone metastases were detected in 3 of the cases [6]. Notably, in our first case, the haematogenous spread to the femur was an early event. Hypercalcemia was also evident in one of our cases, similarly to the observation by Ho et al. and suggests that direct osteolysis in skeletal metastases can be a cause of hypercalcemia in advanced penile cancer, in addition to being a paraneoplastic feature [8]. Thus, the use of bisphosphonates should be considered.

Our observations suggest that the physician should be aware of the possibility for long bone metastases in case of pain in the extremities in patients with penile cancer, even in the early stages of the disease, when there is no known lymphatic spread. Plain X-rays can reveal the skeletal lesion and should be offered early in the course of symptoms. In cases of metastases in the appendicular skeleton, and particularly femoral localisation, surgical treatment is often required in order to alleviate pain associated with weight bearing and to reduce morbidity. This is undoubtedly the case when a pathological fracture is present. Intramedullary nailing can stabilize the fracture but will not prevent progression of the metastasis. Endoprosthetic reconstruction is clearly beneficial in this setting [9]. Adjuvant radiotherapy is always indicated but when the skeletal destruction progresses, en bloc resection and reconstruction of the skeletal defect may be warranted.

In conclusion, thigh pain in patients with penile cancer, especially during ambulation, should alert the treating physician for the presence of a skeletal metastasis. When a pathological fracture has occurred, surgical treatment is necessary, and prosthetic reconstruction is often required.
